# Exploring the origin of a unique mutant allele in twin-tail goldfish using CRISPR/Cas9 mutants

**DOI:** 10.1038/s41598-024-58448-2

**Published:** 2024-04-15

**Authors:** Shu-Hua Lee, Chen-Yi Wang, Ing-Jia Li, Gembu Abe, Kinya G. Ota

**Affiliations:** 1 Laboratory of Aquatic Zoology, Marine Research Station, Institute of Cellular and Organismic Biology, Academia Sinica, Yilan, 26242 Taiwan; 2 Division of Developmental Biology, Department of Functional Morphology, Faculty of Medicine, School of Life Science, Tottori University, Nishi-cho 86, Yonago, 683-8503 Japan

**Keywords:** Developmental biology, Evolution

## Abstract

Artificial selection has been widely applied to genetically fix rare phenotypic features in ornamental domesticated animals. For many of these animals, the mutated loci and alleles underlying rare phenotypes are known. However, few studies have explored whether these rare genetic mutations might have been fixed due to competition among related mutated alleles or if the fixation occurred due to contingent stochastic events. Here, we performed genetic crossing with twin-tail ornamental goldfish and CRISPR/Cas9-mutated goldfish to investigate why only a single mutated allele—*chdS* with a *E127X* stop codon (also called *chdA*^*E127X*^)—gives rise to the twin-tail phenotype in the modern domesticated goldfish population. Two closely related *chdS* mutants were generated with CRISPR/Cas9 and compared with the *E127X* allele in F2 and F3 generations. Both of the CRISPR/Cas9-generated alleles were equivalent to the *E127X* allele in terms of penetrance/expressivity of the twin-tail phenotype and viability of carriers. These findings indicate that multiple truncating mutations could have produced viable twin-tail goldfish. Therefore, the absence of polymorphic alleles for the twin-tail phenotype in modern goldfish likely stems from stochastic elimination or a lack of competing alleles in the common ancestor. Our study is the first experimental comparison of a singular domestication-derived allele with CRISPR/Cas9-generated alleles to understand how genetic fixation of a unique genotype and phenotype may have occurred. Thus, our work may provide a conceptual framework for future investigations of rare evolutionary events in domesticated animals.

Breeders have long been interested in producing ornamental animals with rare phenotypes through artificial selection. Peculiar colorations and morphologies have been genetically fixed in various mammals, birds and teleost species due to the attractiveness of these characteristics to humans^1–11^. Although these ornamental phenotypes may ultimately impair the fitness of the animal in natural conditions, the traits can be fixed in a population due to strong selection pressure applied by breeders (for example, refs^1,8,12^). Recent genetics and genomics studies have identified the responsible alleles for many rare phenotypes in several domesticated animal species^2–11,13^. Intriguingly, some of the identified alleles show polymorphic variations, and for some traits, related mutations can be found in closely related species^6,13,14^. Studies on these alleles have greatly contributed to our understanding of evolutionary processes related to frequently and repeatedly occurring mutations^13^.

In contrast to the ample body of work on frequently occurring mutations, few studies have been conducted on unique genetic mutations. Since singular genetic mutations pose intrinsic challenges for investigations by conventional comparative approaches, little is known about how unique mutated alleles may have come to be fixed in modern populations of domesticated animals. Furthermore, while studies on protein function can often be conducted in fast-replicating, genetically pliable microorganisms^15,16^, investigations on high-order complex phenotypes, such as musculoskeletal morphology, cannot follow this approach. Instead, studies on the high-order complex phenotypes require comparisons of hypothetical ancestral mutant alleles with observed alleles in relatively large multicellular organisms that are slow-growing and not readily amenable to genetic modification. Such limitations have long impeded investigations of the evolutionary process of rare mutated alleles, even though some rare phenotype-associated alleles have been reported in domesticated animals^17^.

Recent advances in genome editing with CRISPR/Cas9 have opened up the possibility of creating almost any desired mutant allele in a wide variety of animal species^18–23^. As such, this technical breakthrough can be applied to enable comparative studies between real and hypothetical alleles in various domesticated animals. In particular, ornamental goldfish (*Carassius auratus*) are especially well-suited for studies on the evolutionary consequences of rare and unique alleles due to the accessibility of their embryos and the highly divergent morphologies observed across different ornamental strains^24^.

Among the menagerie of animals with ornamental phenotypes, the twin-tail ornamental goldfish has particularly intrigued researchers^1,6,7,25–30^. The twin-tail phenotype is known to be caused by a singular mutated allele in the *chordin* gene locus, which is designated as *E127X* or *chdS*^*E127*^^*X*^ in the present study (also known as *chdA*^*E127X*^). This mutation causes the loss of three out of four Cysteine-Rich (CR) domains that are crucial for full function of the encoded protein, restricting the activity of the *chordin* gene product. As a result, animals carrying the *E127X* allele exhibit a ventralized embryonic phenotype that ultimately gives rise to the twin-tail morphology in adult goldfish^4^. Although *chordin* gene mutants in medaka and zebrafish also exhibit twin-tail phenotypes, the mutants are inviable due to their severe or lethal phenotypes^31,32^. Moreover, no similar phenotype has been found in ornamental common carp (*Cyprinus carpio*), even though this teleost species is phylogenetically proximate to goldfish^33^. In short, aside from the twin-tail goldfish with the *E127X* allele of *chdS* gene, there are no known examples of viable vertebrate species with major changes in the function of the *chordin* gene, even among domesticated animals^4,26^.

The uniqueness of the *E127X* allele has been confirmed by multiple genetic and genomic studies^4,6,7^. It is well established that all twin-tail goldfish strains carry the *E127X* allele, and no other related mutated alleles have been identified in the *chdS* locus. Therefore, this singular allele was fixed in the domesticated ornamental goldfish population as a result of intensive selection of the twin-tail phenotype by breeders seeking to establish the ornamental trait^4,6,7^. Two hypotheses can be put forth to explain the conspicuous absence of related polymorphic mutated alleles in the *chdS* locus. One is that the *E127X* allele is somehow advantageous and has outcompeted any other mutated alleles. The other is simply that related polymorphic alleles were absent in the common ancestral population. To determine which of these hypotheses is more likely to be correct, we sought to compare the phenotypes of hypothetical polymorphic mutated alleles with the actual domestication-derived *E127X* allele.

To this end, we used CRISPR/Cas9 to create *chdS* gene mutant alleles and then performed genetic crossing experiments to compare the novel mutants with the *E127X* allele. Unlike the results of previous morphant analyses and insertional mutagenesis studies in goldfish^4,28,29,33,34^, we succeeded in generating F2 and F3 segregants that could be compared in terms of expressivity/penetrance of the twin-tail phenotype and carrier viability on a consistent genetic background. Despite the widespread application of CRISPR/Cas9-based techniques in various vertebrate species, including goldfish, researchers have not yet compared CRISPR/Cas9-generated alleles with traditional domestication-derived alleles in ornamental animals^18–21,23^. Hence, our study is the first to employ genome editing in a study to assess whether a unique mutated allele in an ornamental domesticated animal is more likely to have become singular due to selective pressure or stochastic events.

## Results

**Figure 1:**
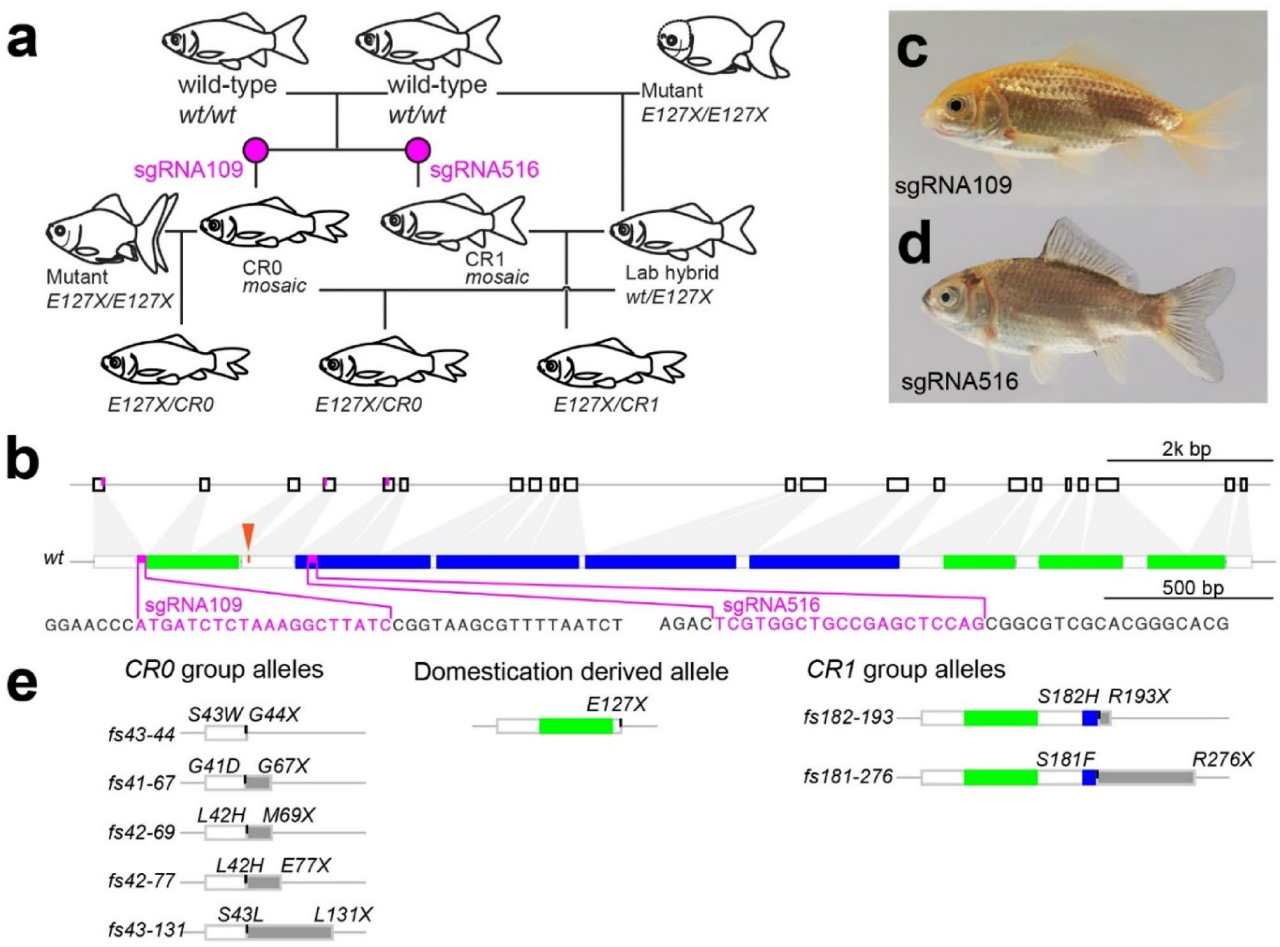
Generation of CRISPR/Cas9-derived *chordin* gene mutant goldfish. (**a**) Experimental procedure for producing CRISPR/Cas9-derived *chordin* mutant goldfish. Magenta letters indicate injected sgRNAs. (**b**) Exon/intron structure, conserved modules, and sgRNA target sites in the *chdS* gene. White boxes indicate exons; green boxes indicate CR domains; blue boxes indicate CHRD domains; magenta boxes indicate sgRNA-targeted sites; and orange arrowhead indicates the position of the *E127X* allele^4,41,44^. (**c**,**d**) Adult specimens of CRISPR/Cas9-mutated goldfish. (**e**) Various alleles of the *chdS* gene used in this study. Black marks indicate mutation sites; grey boxes indicate frame-shifted regions.

To generate mutated alleles related to *E127X*, we designed two sgRNAs (sgRNA109 and sgRNA516) for CRISPR/Cas9-mediated mutagenesis (Fig. 1a). As expected, the sgRNA109- and sgRNA516-injected F0 progenies showed indels respectively in exons 1 and 5 (Fig. 1b; Supplementary Fig S1). The location in exon 1 targeted by sgRNA109 is immediately upstream of the first of four CR domains, which is highly conserved and crucial for the function of the *chordin* gene in dorsal–ventral patterning^31,32,35–40^. The location in exon 5 targeted by sgRNA516 is within the first of four CHRD domains^41^ (Fig. 1b). By co-injecting the sgRNA with Cas9 protein into embryos derived from the single-tail common goldfish parents (*chdS*^*wt/wt*^) (Fig. 1a), we succeeded in producing mosaic F0 progenies with different mutations in the *chdS* gene (Fig. 1c,d). Most of these CRISPR/Cas9-induced mutations caused frameshifts in the *chdS* gene that led to premature truncations and eliminated conserved domains (Fig. 1e; Supplementary Fig. S2). The alleles were therefore categorized as two types (*CR0* and *CR1* type alleles) based on the number of CR domains retained (Fig. 1e; Supplementary Fig. S2; Methods). *CR0* alleles had completely lost all CR domains, while *CR1* alleles retained the first CR domain.

**Figure 2:**
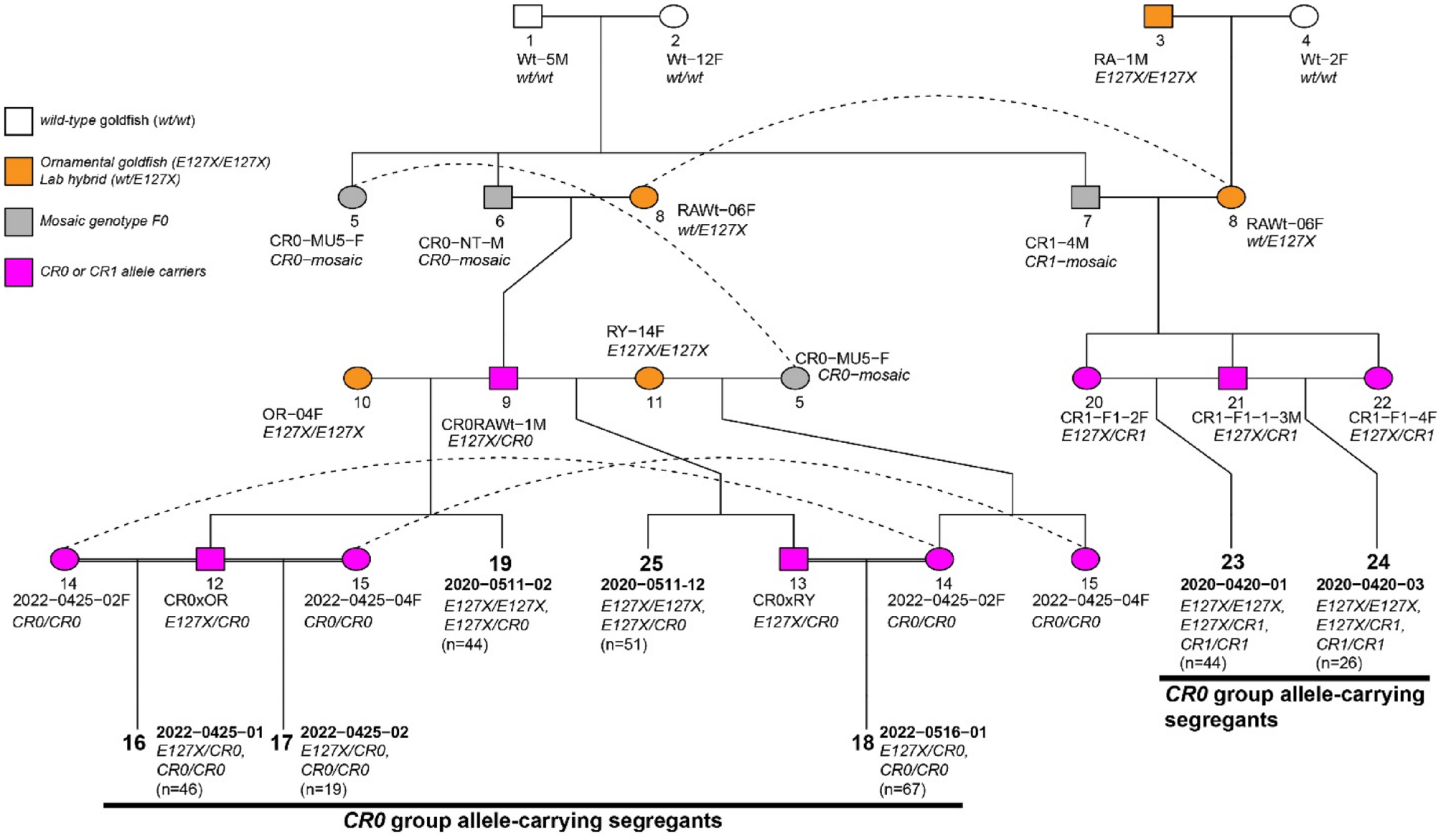
Pedigree of CRISPR/Cas9-derived mutant goldfish strains. White color indicates the wild-type goldfish strains (1, 2 and 4). Orange color represents the individuals derived from an ornamental strain or a hybrid between ornamental goldfish (3, 10 and 11) with *E127X/E127X* genotypes, or a hybrid between ornamental strain and the laboratory wild-type strain (8) with the *wt/E127X* genotype. The CRISPR/Cas9-derived F0 individuals are represented with grey color (5, 6 and 7). Magenta color indicates the F1, F2 and F3 generation individuals carrying CRISPR/Cas9-derived mutant *chdS* alleles. The genotypes are shown in italic letters under the handling ID. The investigated segregant populations are indicated by bold numbers. Allelic combinations of parental CRISPR/Cas9-mutated individuals are as follows: 9, Cr0RAwt-1M (*E127X/fs42-69*); 14, 2022-0425-02F (*fs42-69/fs43-131*); 12, CR0xOR (*E127X/fs42-77*); 15, 2022-0425-04F (*fs42-69/fs43-131*); 13, CR0xRY (*E127X/fs42-77*); 20, CR1-F1-2F (*E127X*/*fs182-193*); 21, CR1-F1-1-3M (*E127X*/*fs182-193*); 22, CR1-F1-4F (*E127X*/*fs182-193*). The nucleotide sequences of the alleles are available in Supplementary Fig. S2.

**Figure 3:**
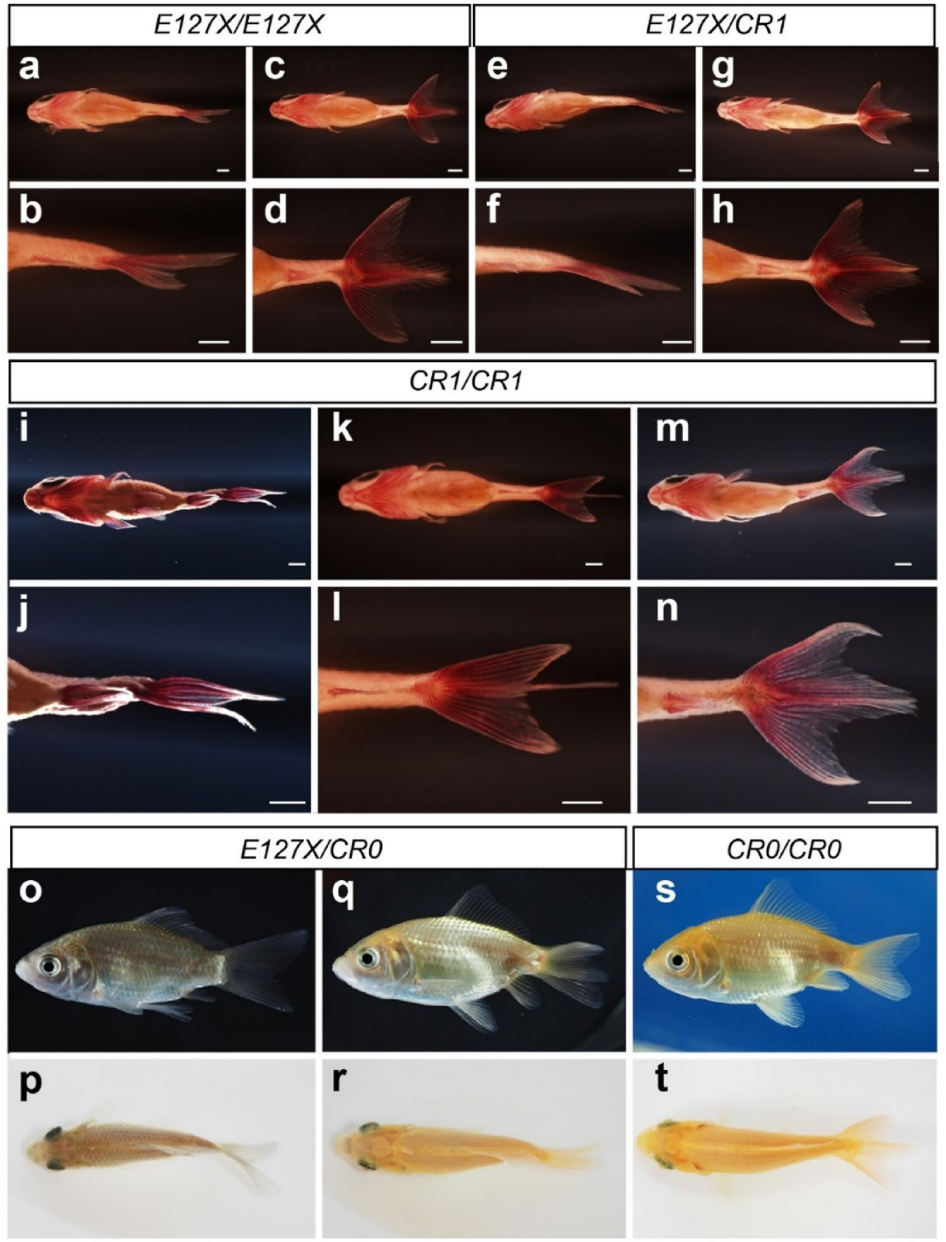
Representative morphologies of F2 progenies. (**a**–**d**) *E127X*/*E127X* genotype progenies. (**e**–**h**) *E127X*/*CR1* genotype progenies. (**i**–**n**) *CR1*/*CR1* genotype progenies. (**o**–**r**) *E127X*/*CR0* genotype progenies. (**s**,**t**) *CR0*/*CR0* progeny. (**a**–**n)** Ventral views of fixed and alizarin red-stained progenies. (**o**–**t**) Lateral view of live progenies. (**a**), (**b**), (**e**), (**f**), (**i**), (**j**), (**o**), (**p**) Single caudal fin progenies. (**c**), (**d**), (**g)**, (**h**), (**k**), (**l**), (**m**), (**n**), (**q**–**t)** Twin-tail progenies. The sizes of the goldfish in panels (**o**), (**q**), and (**s**) are all approximately 5 cm standard length. Scale bars = 1 mm.

To compare *E127X* with *CR0* and *CR1* type alleles under the same genetic background, we obtained F2 and F3 progenies from three F0 CRISPR/Cas9-derived mutant progenies (Fig. 2; Methods; Supplementary Table S1). In total, 297 available specimens were obtained in seven different clutches from the genetic crosses between *CR0*, *CR1,* and *E127X* allele carriers (Fig. 2; Supplementary Data S1). Phenotypes of the F2 and F3 progenies were recorded after the Pelvic fin bud stage in which the anal and caudal fin morphologies are clearly observed (Fig. 3; Supplementary Data S1)^30^. Our morphological observations indicated that each genotype produces different caudal fin morphologies, including both single-tail and twin-tail phenotypes (Fig. 3). Although a few of the twin-tail phenotype goldfish exhibited bifurcation of only the lower fin lobe (Fig. 3k), most exhibited bifurcated morphology of both the upper and lower fin lobes (Fig. 3c,g,m,s). Observations from goldfish with each possible combination of genotype suggested that *CR0* and *CR1* alleles are equivalent to the *E127X* allele in producing a highly bifurcated caudal fin (Fig. 3c,d,g,h,m,n,s,t).

**Figure 4:**
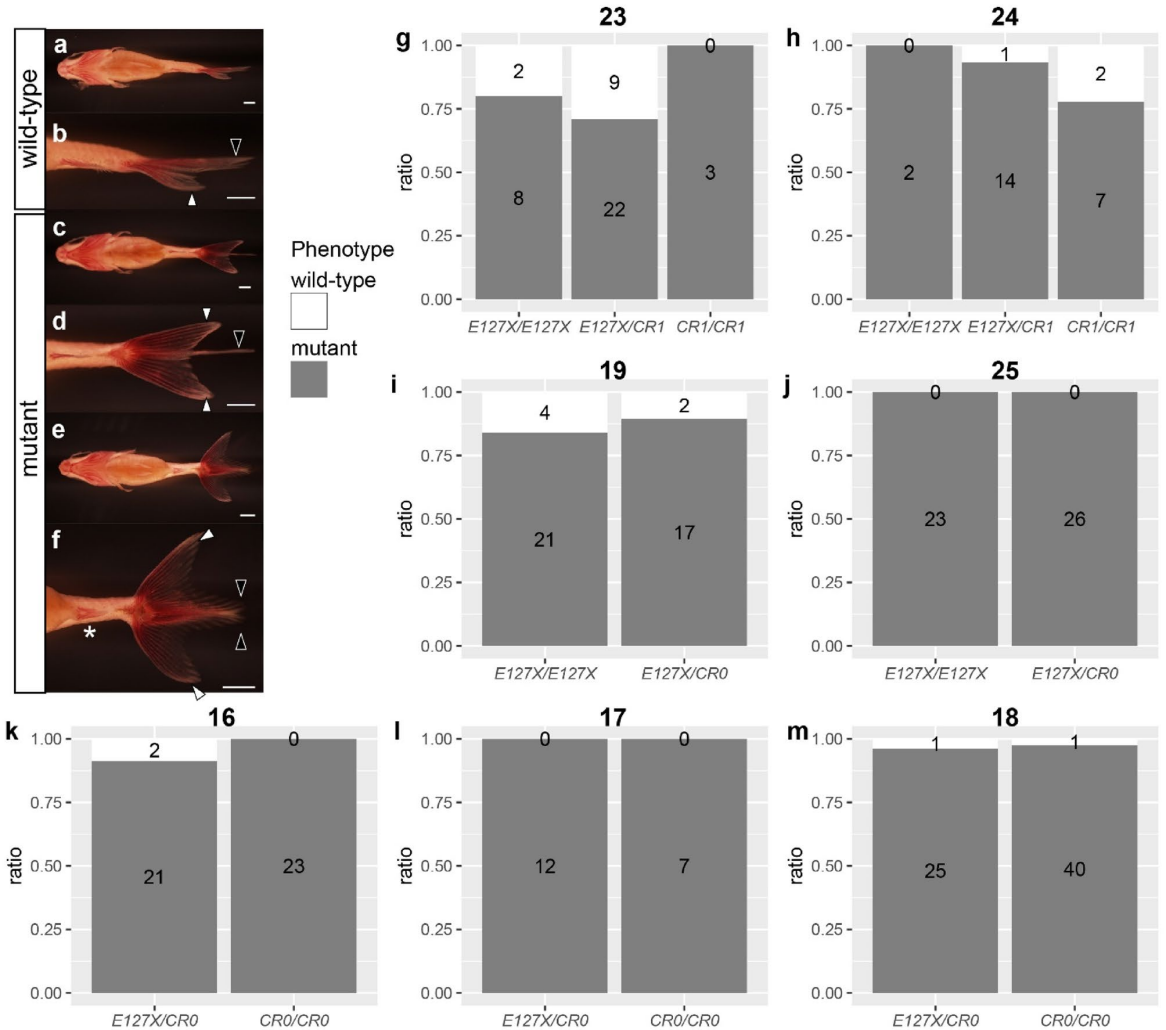
Penetrance of *chdS* mutation alleles. (**a**–**f)** Representative examples of goldfish larvae phenotypes: wild-type (**a**,**b**) and mutants (**c**–**f**). Panels (**a**–**f**) correspond to (**a**), (**b**), (**k**), (**i**), (**g**), and (**h**) in Fig. 3, respectively. White and black arrowheads indicate lower- and upper fin lobes. The duplicated anal fin is indicated by a white asterisk. (**g**–**m**) Proportions of observed caudal fin phenotypes in the F2 generation. The bold numbers above the graph correspond with the numbers in Fig. 2: (**g**) 23, 2020-0420-01; (**h**) 24, 2020-0420-03; (**I**) 19, 2020-0511-02; (**j**) 25, 2020-0511-12; (**k**) 16, 2020–0425-01; (**l**) 17, 2022-0425-02; (**m**) 18, 2022-0516-01. The original dataset is available in Supplementary Data S1. Scale bars (**a**–**f**) = 1 mm.

We further examined the penetrance of mutations in the 297 segregants by categorizing phenotypes into different groups based on caudal and anal fin morphologies (Fig. 4a–f; Methods). Our comparative analysis did not reveal any significant differences between the *E127X* mutant and CRISPR/Cas9-induced alleles in terms of penetrance. Of note, the *CR1*/*CR1* genotype exhibited higher penetrance of the mutated phenotype in one clutch (2020-0420-01), but the other clutch (2020-0420-03) showed an opposite trend for penetrance (clutches 23 and 24 in Fig. 2, Fig. 4g,h). In addition, none of the clutches containing *CR0* alleles had significant differences in penetrance of the mutant phenotype (Fig. 4i–m); all statistical tests yielded p-value exceeding 0.1. Similar to the lack of differences in penetrance, the viability of offspring and expression of bifurcated lower and upper fin lobes were all similar between the different genotypes (Supplementary Fig. S3, S4). In sum, we detected no phenotypic differences in our comparison of *E127X* with *CR0* and *CR1* alleles, despite the different lengths of coding regions (Fig. 1e).

## Discussion

By applying CRISPR/Cas9-mediated genome editing, we were able to compare a unique domestication-derived allele with related hypothetical mutated alleles in goldfish segregants. Based on our comparisons, it was revealed that all of the tested genotypes led to equivalent twin-tail phenotypes (Fig. 3 and 4). More specifically, even when all of the CR domains in *chdS* were deleted, a viable twin-tail goldfish strain could be produced, presumably due to the compensatory function of *chdL* (paralogue of *chdS*, also known as *chdB*) (Fig. 1e and 4)^4^. We further found that a mutant encoding a slightly longer amino acid sequence than that produced by the *E127X* allele can produce the twin-tail phenotype as well (Fig. 1e and 4). These experimental results allow us to identify a specific range in the locus where phenotype-producing stop codon mutations might have been hypothetically retained during domestication; this range spans at least from the 44th amino acid residue to the 276th residue (Fig. 1b).

**Figure 5:**
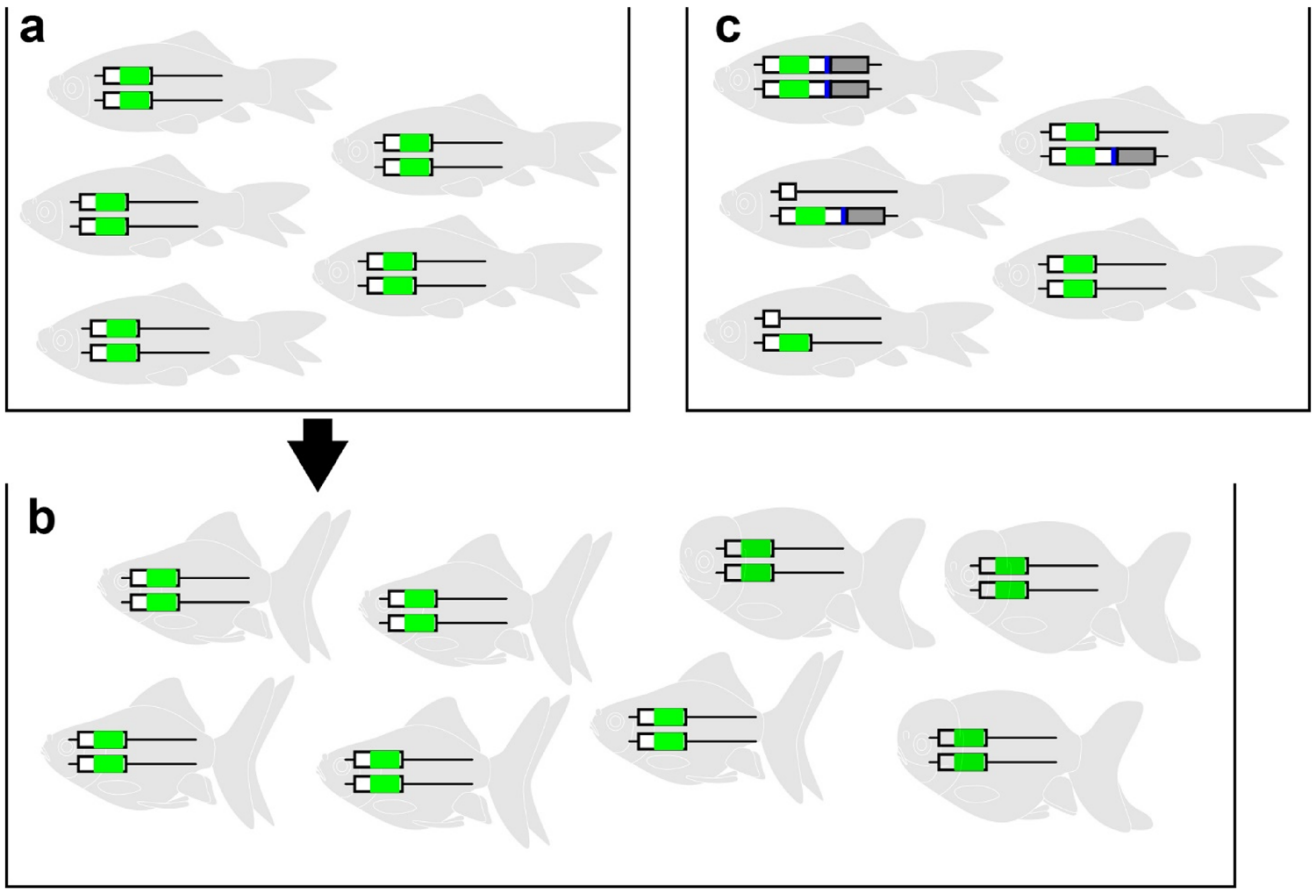
Hypothetical ancestral *chdS* genotypes in twin-tail goldfish populations. (**a**) Hypothetical genotypes at the *chdS* locus in an ancestral twin-tail goldfish population carrying only the *E127X* allele. (**b**) Real modern goldfish population. Alleles are represented by boxes colored with green, blue, white and gray, corresponding with Fig. 1e. (**c**) Hypothetical ancestral genotypes at the *chdS* locus in a twin-tail goldfish population with multiple competing mutated alleles related to *E127X*. The black arrow from (**a**) to (**b**) indicates the most plausible evolutionary scenario according to the findings of this study.

It is still possible that we might detect significant differences between the *E127X* and CRISPR/Cas9-derived *chdS* alleles if the expressed phenotypes were measured at even higher resolution. If we were to examine larger numbers of goldfish at the levels of molecular activity, physiology, developmental biology, microscale morphology, and senescence phenotypes, it is likely that differences would be detected between the alleles. However, taking into account that the primary interest of early Chinese breeders was macroscopic morphology of the goldfish among a limited number of individuals that could be kept in jars and pots, it is reasonable to expect that our results based on visible morphological features are suitable to inform us about the relationship between mutated alleles and phenotypes during the domestication process (Figs. 3 and 4)^42^. Thus, our morphological analyses of F2 and F3 segregants of *E127X* and CRISPR/Cas9 mediated alleles can suggest an answer to the specific question of why similar polymorphisms to *E127X* have not been observed (truncations occurring between amino acid residues 44 to 276) in the modern twin-tail ornamental goldfish population (Fig. 5)^24,42^.

Since the *E127X* allele was not associated with any obvious advantageous characteristic in our study, it seems plausible that only the *E127X* mutation occurred at the *chdS* locus in the ancestral twin-tail goldfish population (Fig. 4, Supplementary Figs S3, S4 and Fig. 5a,b). Our study shows that alternative stop codon mutations related to *CR0* and *CR1* could hypothetically be retained as polymorphic alleles in the modern goldfish population if such mutations had been present in a common ancestor (Fig. 5c). Thus, it is reasonable to assume that the *E127X* allele appeared as a quite rare allele in the common ancestral goldfish *chdS* gene and no competing mutation was present. Alternatively, any competitive alleles (like *CR0* and *CR1* alleles) that arose might have been stochastically eliminated from the population (Fig. 5a,b).

The uniqueness of the *E127X* allele can also be considered by comparing it with known polymorphic alleles causing other phenotypes. For instance, *Mc1r* is responsible for coat color variations in rodents and *MITF* controls spotting patterns in dogs. For both loci, multiple mutant alleles are present in the population, presumably due to the increased fitness conferred by color and pattern variations under different environments^13,14^. Similarly, various mutations in different sites of the *lrp2a1* gene cause telescope-eyes of ornamental goldfish and are retained as polymorphisms^6^. Given that the *bugeye* zebrafish *lrp2* mutant does not have a severe phenotype at the early embryonic stage, and its penetrance is quite low, this gene is expected to have accumulated multiple mutated alleles in the ancestral population of goldfish^43^. In contrast, depletion of the *chordin* gene causes severe phenotypes not only in medaka and zebrafish but also in other vertebrates, including Xenopus and mouse, due to the disruption of dorsal–ventral patterning in early embryonic development^31,32,38,44–47^. Thus, it is highly unlikely that mutated polymorphisms in this gene would be retained in any vertebrate lineage. Even if a similar mutation at the *chdS* locus had occurred and caused a viable twin-tail phenotype in the ancestral *Carassius* species, the mutations would almost certainly not have spread into the population before the Middle Ages when people began to keep ornamental goldfish indoors; the twin-tail phenotype would be extremely disadvantageous to an ancestral goldfish in an open outdoor environment, as twin-tail fish exhibit poor mobility^24,42,48^. Taking into account the high level of evolutionary conservation in the *chordin* gene, it is exceedingly unlikely that various mutated alleles would have existed in the common ancestor of goldfish. This reasoning leads us to conclude that *E127X* was almost certainly the only mutated allele available for early goldfish breeders (Fig. 5a).

Our integrated approach of CRISPR/Cas9 gene editing and crossbreeding experiments with twin-tail goldfish sheds light on how an extremely rare allele that causes a rare phenotype might have evolved in a lineage of domesticated animals. Although we used the ornamental goldfish as a model animal in this study, the same approach can be applied to study other unique mutations that have been identified in various domesticated animals^17,49^. This study presents a strategy for gaining a deeper understanding of why and how a rare allele could be retained without related polymorphisms. Thus, our work may serve as a foundation for future research on rare evolutionary events in domesticated animals.

## Methods

### Goldfish strains

The single-tail and ornamental twin-tail goldfish (Ryukin [RY], Oranda [OR], and Ranchu [RA]) were purchased from an aquarium fish supplier in Taiwan. All single-tail common goldfish parents were genotyped at the *chdS* locus using PCR and restriction enzyme digestion, as described in our previous report^4^. After genotyping the single-tail goldfish, individuals with the *wt/wt* genotype were used for CRISPR/Cas9-mediated genome editing. The nomenclature of goldfish strains is based on our previous reports^24,50^.

### Artificial fertilization

During the spring season (March to June), Ovaprim (Syndel, USA) was injected into goldfish adults to stimulate sperm production in males and to induce spawning by females 12–16 h before artificial fertilization. Sperm was extracted from male goldfish and preserved in Modified Kurokawa’s extender 2 solution at 4 °C^51^. Eggs were squeezed from a mature female goldfish onto PFTE plastic dishes containing tap water. The eggs were bleached with 0.005% sodium hypochlorite for 5 to 10 min, neutralized with 0.5% sodium thiosulfate solution for 1–2 s, and rinsed with tap water. Before placing the eggs in plastic dishes, the bottom of each dish was treated with the green tea beverage “Cha-Li-Wan” (Uni-President Corp., Taiwan) to reduce the occurrence of defects in the egg chorion and to facilitate the detachment of eggs from the plastic dishes^30,52^. Plastic dishes containing fewer than 50 fertilized eggs were maintained at 24 °C until embryos reached the desired stage. The research was performed in accordance with internationally recognized guidelines and ARRIVE guidelines. Ethical approval was granted from the Institutional Animal Care & Utilization Committee of Academia Sinica, Taiwan (ID 19-11-1351).

### In vitro synthesis of sgRNAs and microinjection

CRISPR target sequences were selected using the EnGen TM sgRNA template oligo designer to design target-specific DNA oligos for use with the EnGen TM sgRNA synthesis kit, *S. pyognens* (BioLabs, E3322S). Oligo assembly and in vitro transcription of sgRNAs were conducted in reactions incubated at 37 °C for 30 min. The in vitro transcription template was treated with DNase at 37 °C for 15 min. The RNA product was then purified by precipitation with isopropanol. EnGenTM Cas9 NLS recombinant endonuclease, *S. pyogenes,* was purchased from NEB (#M0646M). The purified RNA and Cas9 NLS recombinant endonuclease were diluted with 0.2 M KCl in nuclease-free water, and Phenol Red (Sigma) was added as an indicator at a final concentration of 0.05%. A microinjector (Eppendorf Femtoget; Eppendorf) was used to co-inject sgRNA and Cas9 into the cytoplasm of one-cell stage embryos. The concentrations of sgRNA and Cas9 were respectively 60 pg and 600 pg per embryo. The sequences of the two sgRNAs (sgRNA109 and sgRNA516) are shown in Fig. 1b.

### Screening of CRISPR/Cas9 genome edited F0 and F1 fish

The CRISPR/Cas9-injected embryos were incubated at 24 °C, and their embryonic/larval phenotypes were examined at 3–4 days post-fertilization. The embryos/larvae were examined under a stereomicroscope (Szx16, Olympus, Japan) and categorized as wild-type or mutant progenies. To prevent the loss of twin-tail progenies due to competition from wild-type progenies, the wild-type and mutated progenies were separately maintained and raised in the aquarium tanks. The separated progenies were maintained until the adult stage and used as F0 parents. To select mosaic F0 progenies for crossing experiments, we performed Sanger sequencing and then isolated candidates with different genotypes of CRISPR/Cas9-edited loci.

From the mosaic genotype F0 individuals, F1 progenies were generated as shown in Fig. 2. Based on their phenotypes, the F1 progenies were separated into wild-type and mutant phenotype progenies, and maintained in different aquarium tanks. The genotypes of these individuals were examined at juvenile and adult stages by the Sanger sequencing (Supplementary Table S1). The F1 progenies with CRISPR/Cas9-induced mutant alleles that included frameshift mutations were then used to generate F2 and F3 progenies (Fig. 2).

### Generation and analyses of F2 and F3 progenies

The F2 progenies were generated from the genetic crosses between F1 individuals with CRISPR/Cas9-mutated alleles, and between the F1 individuals and twin-tail ornamental goldfish (OR and RY strains), as shown in Fig. 2. In total, five clutches were obtained and maintained under similar aquarium conditions. From the five clutches, 165 individuals (2020-0511-02 [n = 44], 2020-0511-12 [n = 51], 2020-0420-01 [n = 44], and 2020-0420-03 [n = 26]) were used for the phenotyping, and four individuals (CR0xOR, CR0xRY, 2022-0425-02F, and 2022-0425-04F) were used to generate the F3 individuals (2022-0425-01 [n = 46], 2022-0425-02 [n = 19], and 2022-0516-01 [n = 67]). The phenotyping and genotyping of these progenies were performed on live animals or following fixation of the individual after the Pr stage (see below). F2 and F3 progenies with simple genotypes consisting of *E127X* and a single CRISPR/Cas9-mutated allele were genotyped by PCR and restriction enzyme digestion based on a previous report^4^. Progenies with more than one CRISPR/Cas9-mutated allele were genotyped by nucleotide sequencing.

### Fish maintenance

Embryos were incubated on plastic dishes (9 cm) and then moved to 10 L aquarium tanks with an overflow system (Wei Feng Corp., Taiwan), according to the general practices for zebrafish mutagenesis experiments. The water in the aquarium system was automatically adjusted to a conductivity of 200–300 μS/cm conductivity, pH 6.5–7.5, and temperature of 24–26 °C. At the late larval stages (more than approximately 1 cm total length), progenies were moved to a 50 L tank. Subsequently, candidate parents of further generations were moved to 500 L tanks in the transgenic aquarium facility at the Marine Research Station, ICOB, Academia Sinica.

Progenies were fed with live food (paramecia and/or brine shrimp) and dry food at least once per day; the type of feed depended on the size of the progenies. Prot-stage larvae were fed with paramecia. After Prot-stage, larvae were mainly fed with brine shrimp and supplemented with paramecia, algae and dry food to minimize the risk of starvation and nutritional deficiency. All of the progenies in the different tanks were maintained under the same feeding, water and light conditions. To prevent developmental delays associated with high population density, fewer than 100 goldfish progenies were maintained in the 10 L, 50 L and 500 L tanks.

### DNA extraction and genotyping

Tissues were preserved in TNES-urea buffer (10 mM Tris–HCl, 0.3 M NaCl, 1% SDS, 10 mM EDTA, and 5 M urea) prior to use for DNA extraction. From the preserved tissues, DNA samples were extracted with phenol/chloroform and ethanol precipitation or DNeasy Blood & Tissue Kits (69504, QIAGEN, USA). The extracted DNA samples were dissolved/eluted in water and used as templates for PCR with the primer set listed in Supplementary Table S2.

Amplified PCR product was used for genotyping by restriction enzyme digestion and/or legacy Sanger sequencing. Quality and amount of DNA fragment were assessed by running the amplified DNA fragment on an agarose gel. Restriction enzyme digestion was performed following a protocol in our previous report^4^. Sanger sequencing was performed in two methods: direct sequencing of the PCR product and a cloning-based sequencing method. For the cloning-based sequencing method, amplified DNA fragments were first cloned into a sequencing vector (pCR™-Blunt II-TOPO™, ThermoFisher Scientific). The vectors with inserted PCR fragments were transformed in the *Escherichia coli*, and more than 10 clones were isolated for sequencing by the Sanger sequencing method.

Genotyping was accomplished by visual inspection of the direct PCR product sequencing chromatograph or by multiple alignment of the sequence derived from the cloning-based sequencing methods. Visual inspection of the chromatographs and generation of multiple alignments were performed using BioEdit and GeneSnap Viewer software.

### Phenotyping and acquisition of DNA samples

After anesthetization with MS222, late juvenile and adult specimens were examined by visual inspection focusing on anal and caudal fins. The fish bodies were inspected under multiple orientations in a water-filled container. Then, tissues from the right-side pectoral fins were removed from the live anesthetized individuals for extraction of DNA.

Late larvae and early juvenile stage progenies were anesthetized with MS222 and then fixed in 4% PFA at room temperature overnight. After fixation, the samples were washed with 70% ethanol and stained with the alizarin red solution (0.1% alizarin red in 70% ethanol)^30,53^. Before observation, the alizarin red-stained specimens were washed with 70% ethanol to reduce the background; progenies were then examined for anal and caudal fin morphology.

Both live and alizarin red-stained progenies were first categorized as wild-type or mutant. Individuals in the mutant group had bifurcated caudal fins and were further categorized based on the particular caudal fin morphology. Subgroups were bifurcated lower fin lobe with single upper fin lobe and bifurcated lower fin lobe with bifurcated upper fin lobe. Single and bifurcated fin lobes were identified based on the presence of bifurcated fin rays. The positions of lower/upper fin lobe were based on the concave point of the caudal fin. Representative phenotypes were photographed under stereomicroscopy (SZX16 with DP80, Olympus, Japan) or with a digital camera (EM1 Mark III with 30 mm Macro lens, Olympus, Japan).

Statistical analyses of the acquired phenotype data were conducted using R with Rstudio. Pearson’s chi-square test was conducted for the data sets from the clutches of 2020-0420-01 and 2020-0420-03. The datasets, organized in a two-by-two table (including 2020-0511-02, 2020-0511-12, 2022-0425-01, 2022-0425-02, and 2022-0516-01), were examined using Fisher’s exact test.

### Supplementary Information

Supplementary Figures.

Supplementary Information 1.

Supplementary Table S1.

Supplementary Table S2.

## Data Availability

The sequence data is deposited in DDBJ with the accession ID (LC775070-LC775088).
