# Supplementary material for: Exploring the origin of a unique mutant allele in twin-tail goldfish using CRISPR/Cas9 mutants
Source: Sci Rep. 2024 Apr 15;14:8716. doi: 10.1038/s41598-024-58448-2 (PMC11018756; doi:10.1038/s41598-024-58448-2)
Supplement: Supplementary file 1 — Supplementary Figures. [file 41598_2024_58448_MOESM1_ESM.docx]

**
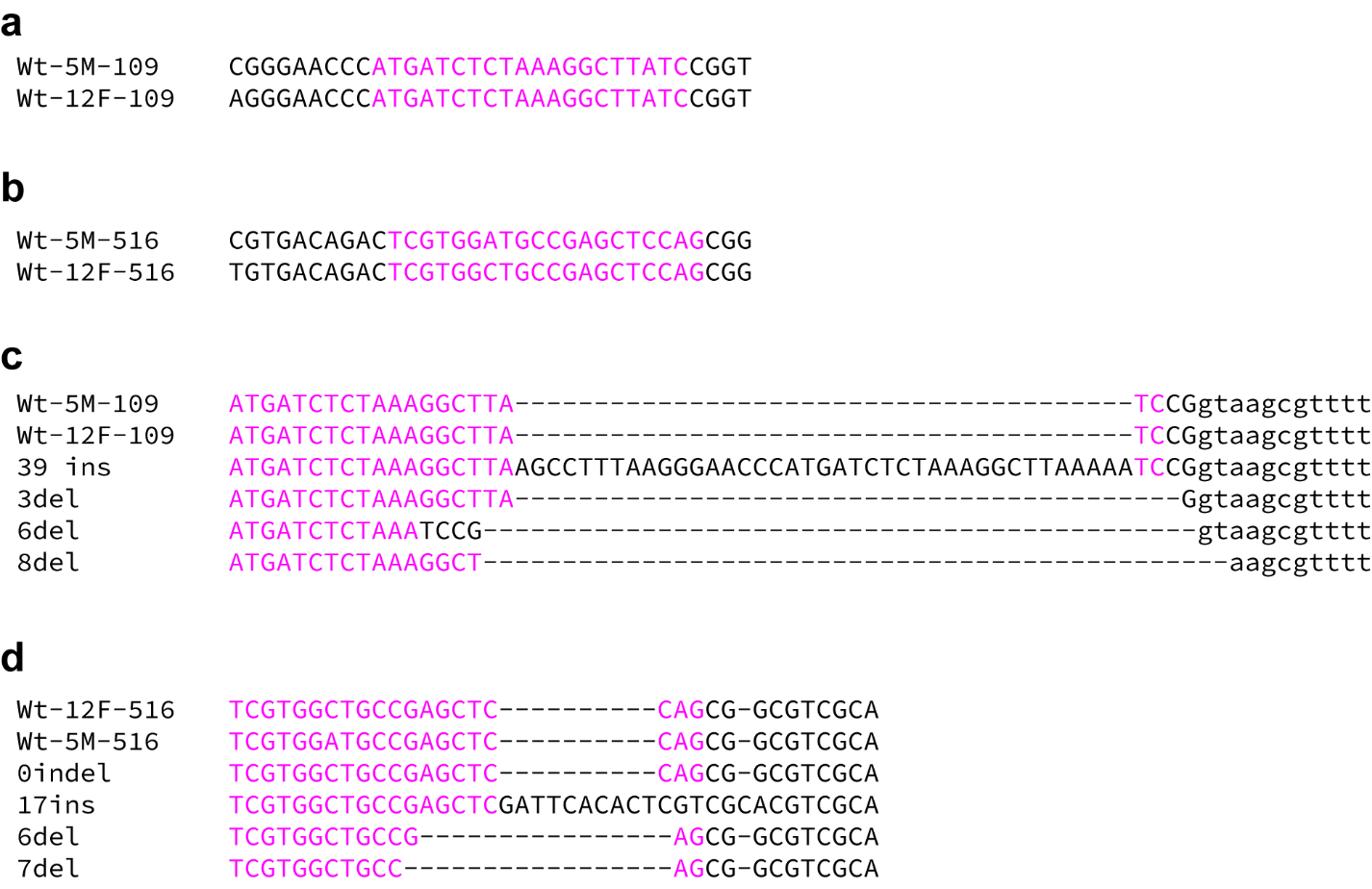
**

## Supplementary Fig. S1: Representative genotyping results for F0 and F1 goldfish

**a**, **b**. Sequences targeted by the sgRNAs in the F0 CRISPR/Cas9-edited goldfish. The magenta letters indicate the sgRNA109-targeted site (**a**) and the sgRNA516-targeted site (**b**). **c**, **d**. Examples of mutations induced by CRISPR/Cas9 genome editing at the sgRNA109 site (**c**) and the sgRNA516 site (**d**). Wt-5M and Wt-12F represent the parents wild-type individual sequences (see **Fig 2**). Accession numbers: Wt-5M-109 (LC775070), Wt-12F-109 (LC775071), Wt-5M-516 (LC775076), Wt-12F-516 (LC775077), 3del-sg109 (LC775072), 6del-sg109 (LC775073), 8del-sg109 (LC775074), 39ins-sg109 (LC775075), 0indel-sg516 (LC775078), 6del-sg516 (LC775079), 7del-sg516 (LC775080), 17ins-sg516 (LC775081).

**
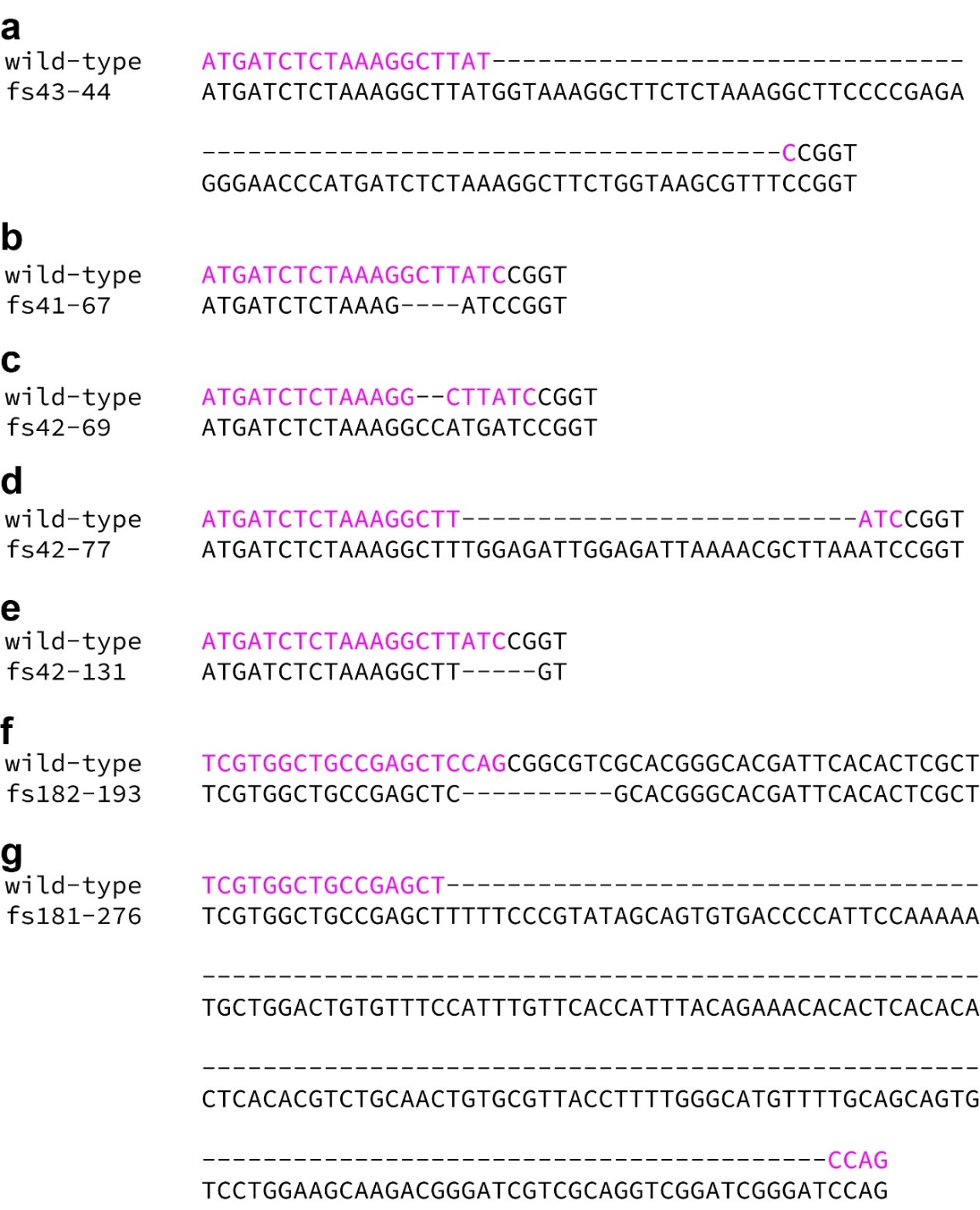
**

## Supplementary Fig. S2: Pairwise comparison between wild-type and CRISPR/Cas9-derived *chdS* alleles

**a**-**e**: CR0 group alleles. **f**, **g**. CR1 group alleles. Magenta letters indicate the region of DNA targeted by the sgRNA. In the allele names, “fs” indicates “frame shift”, while the first and second numbers represent the starting point of the frameshift mutation and the stop codon mutation sites, respectively. Accession numbers: wild-type in panels **a**-**e** (LC775070), wild-type in panels **f**, **g** (LC775076), fs43-44 (LC775082), fs41-67 (LC775083), fs42-69 (LC775084), fs42-77 (LC775085), fs42-131 (LC775086), fs182-193 (LC775087), fs181-276 (LC775088).

**
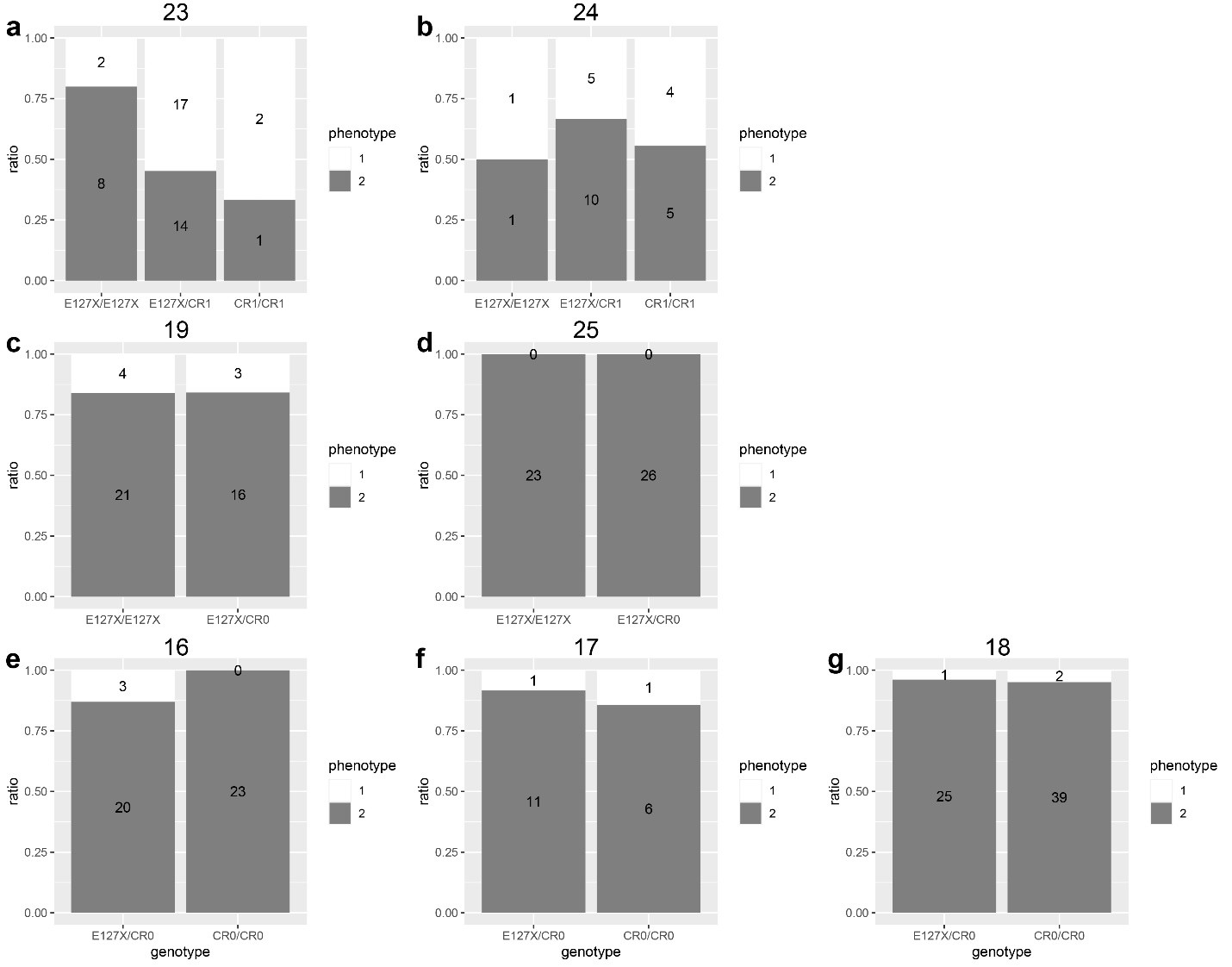
**

## Supplementary Fig. S3: Expressivity of the caudal fin phenotype of bifurcated upper fin lobe

**A**, **B**. CR1 group allele-carrying segregants. **C**-**G**. CR0 group allele-carrying segregants. The phenotype numbers 1 and 2 respectively indicate single and bifurcated upper fin lobes. Statistical comparisons of the ratios between different genotypes did not show any statistically significant differences. The bold numbers above each graph correspond to the numbers in **Figs. 2** and **4**, as follows; **a**. **23**; 2020-0420-01. **b**. **24**; 2020-0420-03. **c**. **19**, 2020-0511-02, **d**. **25**; 2020-0511-12. **e**. **16**; 2020-0425-01. **f**. **17**, 2022-0425-02. **g**. **18**, 2022-0516-01. The original dataset is provided as **Supplementary Data S1**.

**
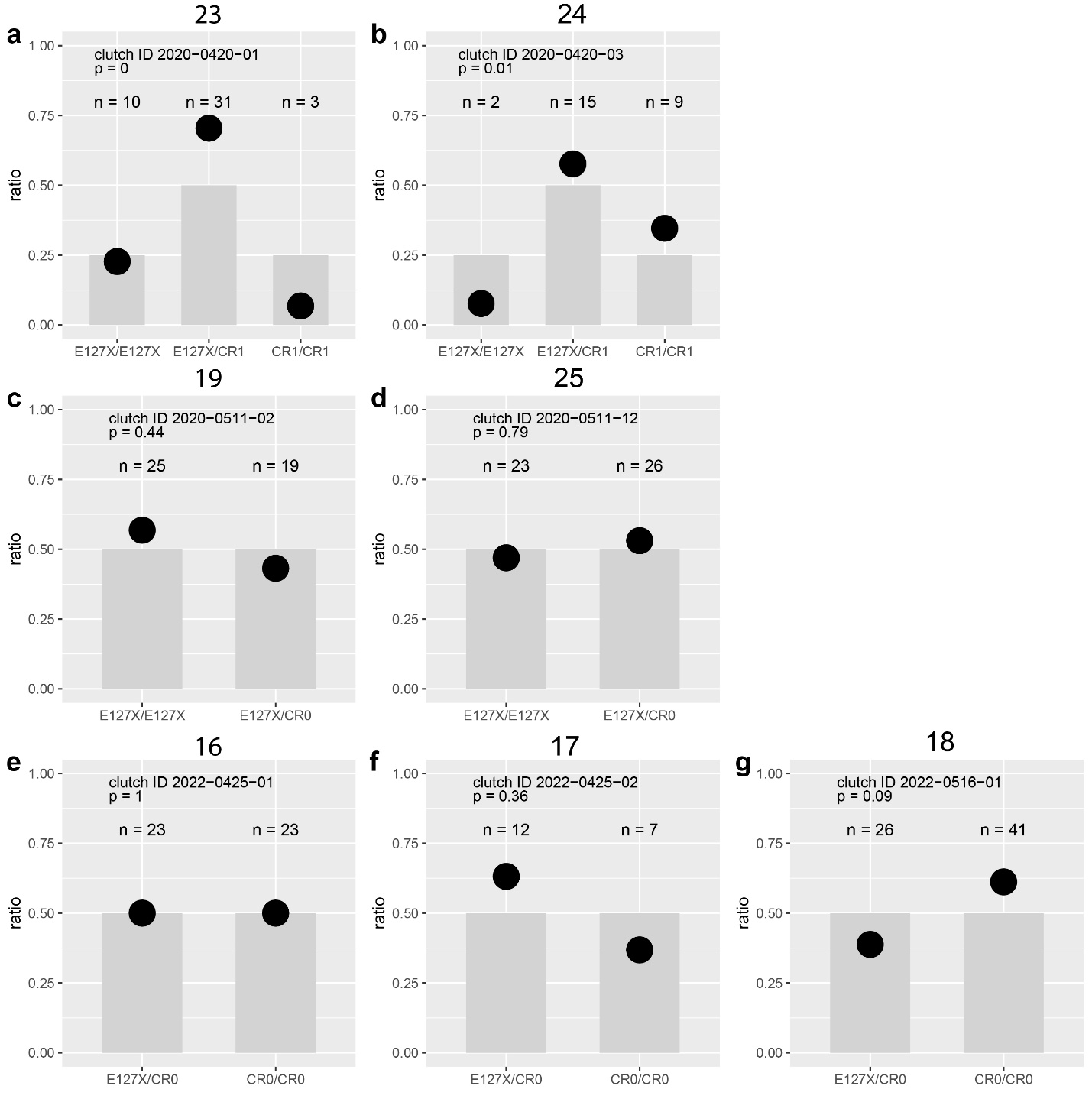
**

## Supplementary Data Fig. S4: Ratios of genotypes at juvenile stage in F2 generation

**a**, **b**. CR1 group allele-carrying goldfish segregants. **c**-**g**. CR0 group allele-carrying goldfish segregants. Statistical significance was calculated by Chi-squared test (**a**, **b**) and Fisher’s exact test (**c**-**g**). Every individual in the same clutch of juveniles was fixed at the same day post-fertilization. The bold numbers above each graph correspond to the numbers in **Figs. 2** and **4**, as follows; **a**. **23**; 2020-0420-01. **b**. **24**; 2020-0420-03. **c**. **19**, 2020-0511-02, **d**. **25**; 2020-0511-12. **e**. **16**; 2020-0425-01. **f**. **17**, 2022-0425-02. **g**. **18**, 2022-0516-01. The original dataset is provided as **Supplementary Data S1**.
